# Supplementary material for: Early forecasting of tsunami inundation from tsunami and geodetic observation data with convolutional neural networks
Source: Nat Commun. 2021 Apr 15;12:2253. doi: 10.1038/s41467-021-22348-0 (PMC8050057; doi:10.1038/s41467-021-22348-0)
Supplement: Supplementary file 1 — Supplementary Information [file 41467_2021_22348_MOESM1_ESM.pdf]

Supplementary information

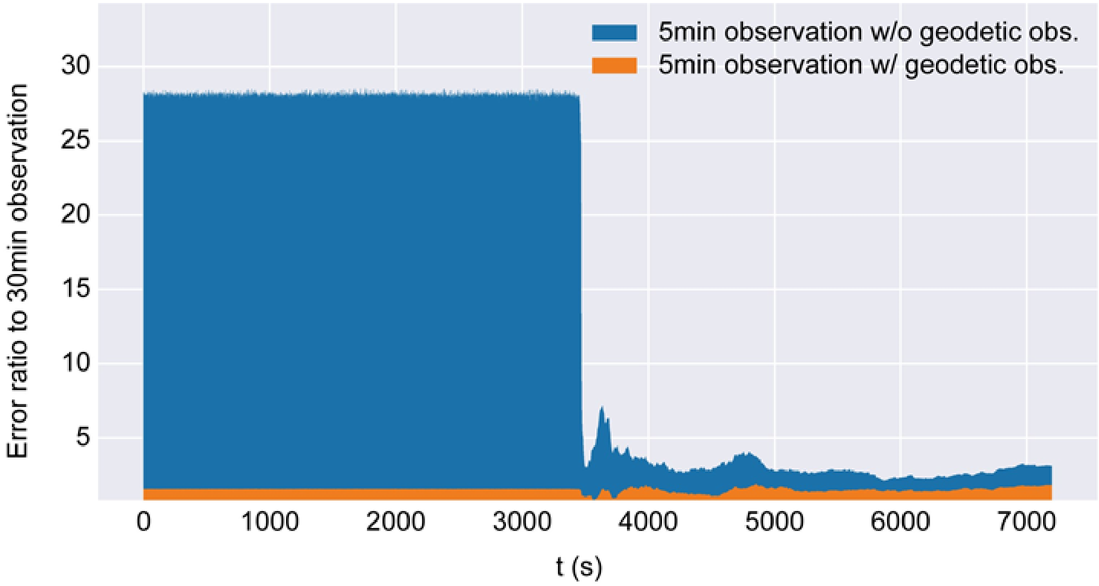

Supplementary Figure 1: Error distribution of 5-min observation models with and without geodetic observation data for a sufficiently long (30 min) observation model. The squared error at each time Et in the prediction was summed over 1,000 test scenarios. The change in error was then visualized as the ratio of Et to the error for a 30 min observation Et_{30}, i.e., Et/Et_{30}. For the 5 min observation model without geodetic observation data, significant errors were observed in the early phase of tsunami inundation, which corresponds to the initial ground height prediction. By inputting geodetic observations, even short-term offshore observations, the CNN can accurately estimate tsunamis.

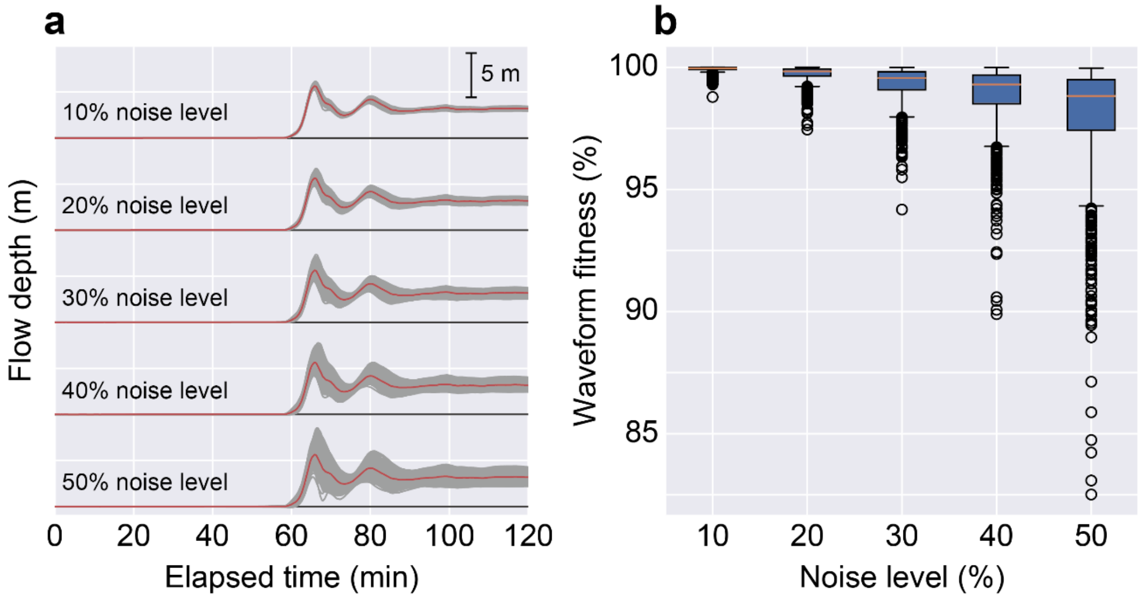

Supplementary Figure 2: Results of the noise tolerance test with random noise. a: CNN forecasting results with different noise levels. Grey lines represent the forecasting results of 1,000 test scenarios with random noise. Res lines are the reference line (forecasting result without noise). b: Distribution of the waveform fitness between the forecasted waveforms with and without noise. The centre lines, box limits, whiskers and points represent median, lower and upper quartiles, 1.5 x interquartile range and outliers, respectively.

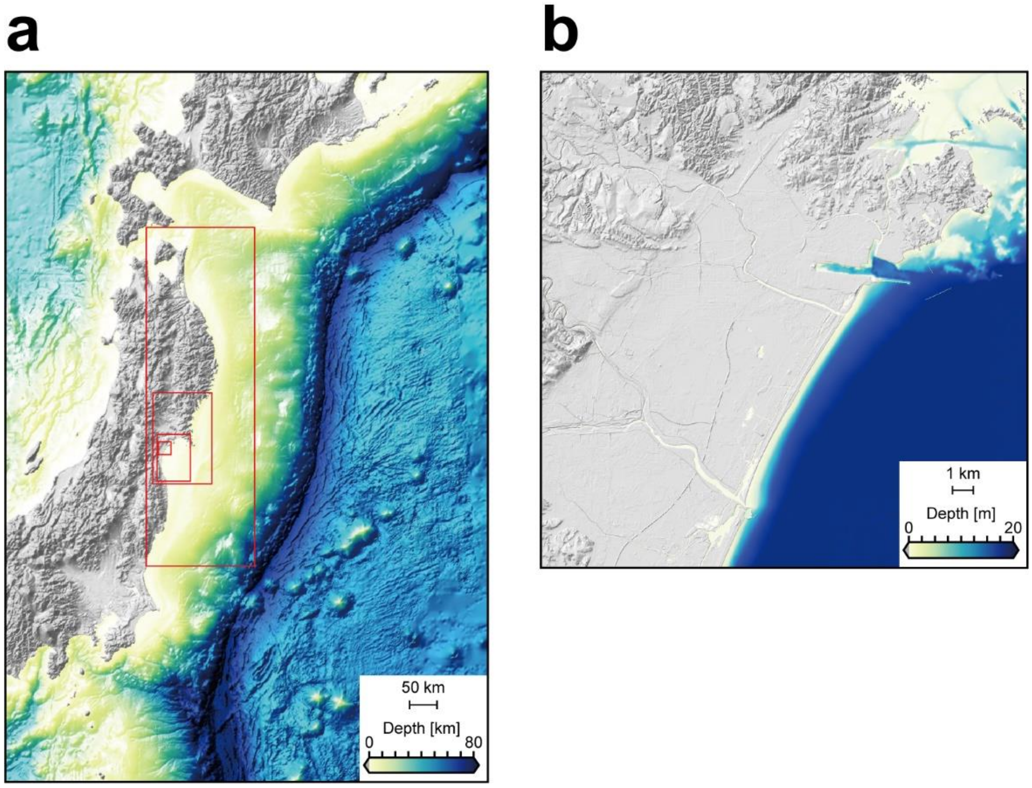

Supplementary Figure 3: Overview of the tsunami simulation settings. a: The tsunami simulation domain. The areas of the nested simulation domains are shown in red rectangles. b: The finest nested domain (Δx = 15 m) for the Sendai Plain, which experienced devastating damage during the 2011 tsunami.

Supplementary Table 1: Architecture of the CNN. A consistent network structure is used for different observation periods. For 5, 15, 25, 35 min observation models, the kernel size of conv3-4 layer was set as 3. For the other CNN models, the kernel size of the conv3-4 was set as 2.

Layer	Kernel size	Stride	Padding	In channels	Out channels
conv1-1	3	1	1	# of observation channels	128
conv1-2	3	1	1	128	128
conv1-3	3	1	1	128	128
conv1-4	4	4	0	128	128
conv2-1	3	1	1	128	128
conv2-2	3	1	1	128	128
conv2-3	3	1	1	128	128
conv2-4	3	3	0	128	128
conv3-1	3	1	1	128	256
conv3-2	3	1	1	256	256
conv3-3	3	1	1	256	256
conv3-4	2 (3)	2	0	256	256
FC1	None	None	None	# of flattened elements	4,096
FC2	None	None	None	4,096	4,096
FC3	None	None	None	4,096	3,600
